# Genetic risk score for risk prediction of diabetic nephropathy in Han Chinese type 2 diabetes patients

**DOI:** 10.1038/s41598-019-56400-3

**Published:** 2019-12-27

**Authors:** Li-Na Liao, Tsai-Chung Li, Chia-Ing Li, Chiu-Shong Liu, Wen-Yuan Lin, Chih-Hsueh Lin, Chuan-Wei Yang, Ching-Chu Chen, Chiz-Tzung Chang, Ya-Fei Yang, Yao-Lung Liu, Huey-Liang Kuo, Fuu-Jen Tsai, Cheng-Chieh Lin

**Affiliations:** 1 Department of Public Health, College of Public Health, China Medical University, Taichung, Taiwan; 2 Department of Healthcare Administration, College of Medical and Health Sciences, Asia University, Taichung, Taiwan; 3 School of Medicine, College of Medicine, China Medical University, Taichung, Taiwan; 4 Department of Medical Research, China Medical University Hospital, Taichung, Taiwan; 5 Department of Family Medicine, China Medical University Hospital, Taichung, Taiwan; 6 Division of Endocrinology and Metabolism, Department of Medicine, China Medical University Hospital, Taichung, Taiwan; 7 School of Chinese Medicine, College of Chinese Medicine, China Medical University, Taichung, Taiwan; 8 Kidney Institute and Division of Nephrology, Department of Internal Medicine, China Medical University Hospital, Taichung, Taiwan; 9 Graduate Institute of Clinical Medical Science, College of Medicine, China Medical University, Taichung, Taiwan; 10 Human Genetic Laboratory, Department of Medical Research, China Medical University Hospital, Taichung, Taiwan

**Keywords:** Chronic kidney disease, Risk factors

## Abstract

We evaluated whether genetic information could offer improvement on risk prediction of diabetic nephropathy (DN) while adding susceptibility variants into a risk prediction model with conventional risk factors in Han Chinese type 2 diabetes patients. A total of 995 (including 246 DN cases) and 519 (including 179 DN cases) type 2 diabetes patients were included in derivation and validation sets, respectively. A genetic risk score (GRS) was constructed with DN susceptibility variants based on findings of our previous genome-wide association study. In derivation set, areas under the receiver operating characteristics (AUROC) curve (95% CI) for model with clinical risk factors only, model with GRS only, and model with clinical risk factors and GRS were 0.75 (0.72–0.78), 0.64 (0.60–0.68), and 0.78 (0.75–0.81), respectively. In external validation sample, AUROC for model combining conventional risk factors and GRS was 0.70 (0.65–0.74). Additionally, the net reclassification improvement was 9.98% (*P* = 0.001) when the GRS was added to the prediction model of a set of clinical risk factors. This prediction model enabled us to confirm the importance of GRS combined with clinical factors in predicting the risk of DN and enhanced identification of high-risk individuals for appropriate management of DN for intervention.

## Introduction

Diabetic nephropathy (DN) is a serious complication in type 2 diabetes patients. Diabetic patients have a more rapid decline in renal function than individuals without diabetes^1^. Without particular managements or specific interventions, approximately 20–40% of patients who have type 2 diabetes and microalbuminuria will progress to macroalbuminuria^2^. Based on the 2017 USRDS reports, diabetes is the primary cause of end-stage renal disease (ESRD) in incident patients; approximately 44–66% of patients with new ESRD in Japan, the USA, Taiwan, South Korea, and Singapore are due to diabetes^3^. A collaborative meta-analysis of general population cohorts revealed that decreased estimated glomerular filtration rate (eGFR) and albuminuria are associated with all-cause and cardiovascular mortality^4^. Furthermore, DN patients have an increased risk of cardiovascular morbidity and mortality^5,6^.

Many prediction models for diseases have been published and widely used. Prediction models can help screen high-risk individuals and assist medical decision-making and health education. Several studies have established risk prediction models considering traditional clinical factors for chronic kidney disease (CKD) or ESRD in the general population^7–12^ or type 2 diabetes patients^13–18^, based on cross-sectional or longitudinal studies. The pathogenesis of CKD appears to be complicated and multifactorial^19^. Macisaac *et al*. reviewed literatures and found that hyperglycemia and predisposing genes are the initiators of diabetic kidney disease^20^. Therefore, using susceptible genes as a predictor only or adding genetic information into a traditional prediction model may be helpful to improve the predictive ability of CKD or ESRD.

A single genetic risk score (GRS), aggregating multiple single-nucleotide polymorphism (SNP) information into a variable, is a useful tool for examining the cumulative predictive ability of genetic variation at known loci on a disease^21^. Khera *et al*. used polygenic scores to quantify inherited susceptibility for common diseases, such as coronary artery disease and obesity, and found that these polygenic scores can identify individuals with risk equivalent to monogenic mutations^22,23^. Moreover, several disease prediction models adding genetic information are being continuously developed, such as those for type 2 diabetes^24^, cardiovascular outcomes^21^, and fracture^25,26^. However, studies using additional genetic information into these clinical risk prediction models of CKD in type 2 diabetes patients are still limited. So far we have found four existing models for predicting CKD risk, including three in the general population^27–29^ and one in type 2 diabetes patients^30^. According to the issues discussed above, it is worth to develop GRS scores based on our prior findings of genome-wide association study (GWAS)^31^. In this study, we evaluated whether genetic information would offer improvement on DN risk prediction upon the addition of susceptibility SNPs identified from our prior GWAS findings^31^ to clinical risk factors in Han Chinese population with type 2 diabetes.

## Results

The characteristics of 995 type 2 diabetes patients in the derivation set and 519 patients in the validation set are summarized in Table 1. A total of 246 (24.7%) DN cases were in the derivation set, while 179 (34.5%) were in the validation set. The mean age for DN and non-DN was 57.54 and 64.32 years, respectively, in the derivation set and 69.73 and 70.55 years, respectively, in the validation set. In both sets, approximately half of the type 2 diabetes patients were male. The proportions of obesity, abnormal triglycerides, hypertension, heart disease, and CVA were higher in the DN cases group than in the control group in both derivation and validation sets. In the derivation set, DN cases had higher percentage of diabetes durations ≥10 years than the control group (56.50% vs. 36.98%). The average GRSs (the number of risk alleles carried) of DN cases and diabetic controls were 2.87 and 2.08 risk alleles, respectively, in the derivation set and 2.49 and 2.28 risk alleles, respectively, in the validation set.

**Table 1:** Demographic and clinical characteristics of study samples.

Characteristic	Derivation sample			Validation sample		
	DN cases (n = 246)	Controls (n = 749)	*P*-value	DN cases (n = 179)	Controls (n = 340)	*P*-value
Age (years)	64.32 ± 9.43	57.54 ± 9.92	<0.01 × 10^−12^	70.55 ± 12.40	69.73 ± 7.12	0.417
Gender			0.813			0.571
Women	124 (50.41)	369 (49.27)		83 (46.37)	168 (49.41)	
Men	122 (49.59)	380 (50.73)		96 (53.63)	172 (50.59)	
Smoking status			0.167			0.472
No	209 (84.96)	605 (80.77)		156 (88.14)	308 (90.59)	
Yes	37 (15.04)	144 (19.23)		21 (11.86)	32 (9.41)	
Alcohol drinking			0.044			0.645
No	206 (83.74)	580 (77.44)		156 (87.64)	304 (89.41)	
Yes	40 (16.26)	169 (22.56)		22 (12.36)	36 (10.59)	
Durations of diabetes	12.11 ± 8.15	8.26 ± 6.62	7.47 × 10^−11^	12.66 ± 10.97	—	—
<10 years	107 (43.50)	472 (63.02)	1.09 × 10^−7^	79 (44.13)	—	—
≥10 years	139 (56.50)	277 (36.98)		100 (55.87)	—	
BMI	25.75 ± 3.94	24.95 ± 3.75	0.004	26.24 ± 4.27	24.92 ± 3.49	0.001
<27 kg/m^2^	150 (60.98)	536 (71.56)	0.002	112 (62.57)	256 (75.29)	0.003
≥27 kg/m^2^ (obesity)	96 (39.02)	213 (28.44)		67 (37.43)	84 (24.71)	
HbA1c	8.15 ± 1.65	7.88 ± 1.42	0.022	7.16 ± 1.59	—	—
<7%	61 (24.80)	209 (27.90)	0.385	97 (56.73)	—	—
≥7%	185 (75.20)	540 (72.10)		74 (43.27)	—	
Creatinine	1.39 ± 1.10	0.73 ± 0.18	<0.01 × 10^−12^	2.89 ± 3.16	0.81 ± 0.18	<0.01 × 10^−12^
Normal (M: 0.7–1.5; F: 0.5–1.2 mg/dL)	183 (74.39)	703 (93.86)	<0.01 × 10^−12^	65 (36.31)	319 (93.82)	<0.01 × 10^−12^
Abnormal	63 (25.61)	46 (6.14)		114 (63.69)	21 (6.18)	
Uric acid (mg/dL)	7.35 ± 1.87	5.87 ± 1.53	<0.01 × 10^−12^	6.88 ± 4.04	5.63 ± 1.34	2.40 × 10^−4^
Normal (M: <7; F: <6 mg/dL)	76 (30.89)	518 (69.16)	<0.01 × 10^−12^	81 (52.94)	255 (75.00)	1.94 × 10^−6^
Abnormal	170 (69.11)	231 (30.84)		72 (47.06)	85 (25.00)	
BUN	24.87 ± 12.38	14.98 ± 4.12	<0.01 × 10^−12^	38.26 ± 27.24	13.61 ± 3.87	<0.01 × 10^−12^
Normal (7–20 mg/dL)	106 (43.09)	659 (87.98)	<0.01 × 10^−12^	40 (28.37)	319 (93.82)	<0.01 × 10^−12^
Abnormal	140 (56.91)	90 (12.02)		101 (71.63)	21 (6.18)	
Total cholesterol	192.90 ± 52.76	186.40 ± 37.14	0.071	171.20 ± 39.63	186.40 ± 35.41	1.04 × 10^−5^
Normal	159 (64.63)	507 (67.69)	0.420	133 (75.57)	230 (67.65)	0.077
Abnormal (≥200 mg/dL)	87 (35.37)	242 (32.31)		43 (24.43)	110 (32.35)	
Triglycerides	189.90 ± 157.6	155.30 ± 117.4	0.002	178.80 ± 153.9	138.50 ± 90.44	0.001
Normal	120 (48.98)	456 (61.13)	0.001	101 (56.42)	227 (66.76)	0.026
Abnormal (≥150 mg/dL)	125 (51.02)	290 (38.87)		78 (43.58)	113 (33.24)	
LDL–C	118.50 ± 42.42	118.10 ± 34.39	0.907	96.26 ± 36.18	113.30 ± 30.76	6.74 × 10^−6^
Normal	167 (67.89)	486 (64.89)	0.434	103 (83.74)	246 (73.87)	0.037
Abnormal (≥130 mg/dL)	79 (32.11)	263 (35.11)		20 (16.26)	87 (26.13)	
HDL–C	46.80 ± 13.75	49.46 ± 13.79	0.009	—	42.98 ± 11.28	—
Normal	131 (53.25)	454 (60.70)	0.047	—	128 (37.65)	—
Abnormal (M: <40; F: <50 mg/dL)	115 (46.75)	294 (39.30)		—	212 (62.35)	
Hypertension			<0.01 × 10^−12^			1.11 × 10^−8^
No	84 (34.15)	457 (61.01)		30 (16.76)	143 (42.06)	
Yes	162 (65.85)	292 (38.99)		149 (83.24)	197 (57.94)	
Heart disease			2.01 × 10^−6^			6.96 × 10^−5^
No	174 (70.73)	634 (84.65)		115 (64.25)	274 (80.59)	
Yes	72 (29.27)	115 (15.35)		64 (35.75)	66 (19.41)	
CVA			3.24 × 10^−4^			1.13 × 10^−6^
No	226 (91.87)	729 (97.33)		135 (75.42)	311 (91.47)	
Yes	20 (8.13)	20 (2.67)		44 (24.58)	29 (8.53)	

Data are presented as mean ± SD for continuous variables or n (%) for categorical variables.

BMI: body mass index; BUN: blood urea nitrogen; CVA: cerebral vascular accident.

The genotype and allele distributions of the study subjects stratified by sample set and DN status are presented in Table 2. The minor allele frequencies in the derivation set (ranges: 0.23–0.48 in DN cases and 0.15–0.36 in controls) were similar with the validation set (ranges: 0.19–0.45 in DN cases and 0.14–0.37 in controls).

**Table 2:** Genotype and allele distributions of study subjects.

SNP	Chr.	Gene	Genotype or allele	Derivation sample		Validation sample	
				DN cases (n = 246)	Controls (n = 749)	DN cases (n = 179)	Controls (n = 340)
rs10963767	9	ADAMTSL1	TT	95 (38.62)	388 (51.80)	83 (46.37)	143 (42.06)
			CT	112 (45.53)	296 (39.52)	82 (45.81)	169 (49.71)
			CC	39 (15.85)	65 (8.68)	14 (7.82)	28 (8.24)
			C*	0.39	0.28	0.31	0.33
rs11647932	16	ST3GAL	CC	145 (58.94)	538 (71.83)	116 (64.80)	253 (74.41)
			TC	87 (35.37)	192 (25.63)	58 (32.40)	79 (23.24)
			TT	14 (5.69)	19 (2.54)	5 (2.79)	8 (2.35)
			T*	0.23	0.15	0.19	0.14
rs11645214	16	SF3B3	AA	63 (25.61)	309 (41.26)	51 (28.49)	129 (38.39)
			GA	130 (52.85)	341 (45.53)	96 (53.63)	163 (48.51)
			GG	53 (21.54)	99 (13.22)	32 (17.88)	44 (13.10)
			G*	0.48	0.36	0.45	0.37
rs6499323	16	IL34	AA	64 (26.12)	327 (43.89)	56 (31.46)	141 (41.72)
			GA	138 (56.33)	323 (43.36)	97 (54.49)	163 (48.22)
			GG	43 (17.55)	95 (12.75)	25 (14.04)	34 (10.06)
			G*	0.46	0.34	0.41	0.34
rs182784	20	BMP7	AA	123 (50.00)	439 (58.69)	95 (53.07)	185 (54.57)
			GA	95 (38.62)	273 (36.50)	73 (40.78)	126 (37.17)
			GG	28 (11.38)	36 (4.81)	11 (6.15)	28 (8.26)
			G*	0.31	0.23	0.27	0.27
rs4811839	20	RAE1	TT	109 (44.31)	420 (56.07)	81 (45.25)	179 (52.80)
			GT	104 (42.28)	281 (37.52)	85 (47.49)	128 (37.76)
			GG	33 (13.41)	48 (6.41)	13 (7.26)	32 (9.44)
			G*	0.35	0.25	0.31	0.28
rs6025517	20	RAE1	TT	115 (46.75)	434 (57.94)	87 (48.60)	185 (54.73)
			CT	103 (41.87)	273 (36.45)	80 (44.69)	123 (36.39)
			CC	28 (11.38)	42 (5.61)	12 (6.70)	30 (8.88)
			C*	0.32	0.24	0.29	0.27

All *P* > 0.05 from Hardy–Weinberg equilibrium test.

Chr.: chromosome. *Minor allele.

Table 3 shows the ORs and their 95% CIs for DN in three models from the derivation sample. We found that age, obesity, abnormal triglycerides, hypertension, and heart disease were significant predictors of DN risk in model 1, and the ORs ranged from 1.07 to 2.03. The crude OR (95% CI) for DN was 1.22 (1.15–1.29) per risk allele of GRS (model 2). After adding GRS into model 1, the risk of DN increased by 1.24-fold (95% CI: 1.17–1.32) for every additional risk allele of GRS. Results of using the weighted GRS (wGRS) as a predictor were presented in Supplement Table 1. The crude and adjusted OR (95% CI) were 1.42 (1.28–1.56) and 1.46 (1.31–1.63) for every one unit increase of wGRS, respectively. Furthermore, we also performed the same analysis by using BMI and triglycerides as quantitative variables. We found that the GRS had the same effect on DN and its OR (95% CI) was 1.24 (1.17–1.32) (Supplement Table 2).

**Table 3:** ORs and their 95% CIs for diabetic nephropathy in derivation sample by using the GRS as predictor.

Variable	Model 1		Model 2		Model 3	
	OR (95% CI)	*P*-value	OR (95% CI)	*P*-value	OR (95% CI)	*P*-value
Gender (ref. women)	1.11 (0.81, 1.51)	0.529	—	—	1.14 (0.82, 1.57)	0.432
Age (years)	1.07 (1.05, 1.09)	<0.01 × 10^−12^	—	—	1.08 (1.06, 1.10)	<0.01 × 10^−12^
Obesity (ref. BMI<27 kg/m^2^)	1.59 (1.14, 2.22)	0.007	—	—	1.61 (1.14, 2.28)	0.007
Abnormal triglycerides (ref.<150 mg/dL)	1.63 (1.19, 2.24)	0.002	—	—	1.56 (1.13, 2.17)	0.008
Hypertension (ref. No)	2.03 (1.46, 2.81)	2.26 × 10^−5^	—	—	2.12 (1.51, 2.98)	1.33 × 10^−5^
Heart disease (ref. No)	1.56 (1.08, 2.26)	0.018	—	—	1.48 (1.01, 2.18)	0.046
GRS (per risk allele)	—	—	1.22 (1.15, 1.29)	9.24 × 10^−12^	1.24 (1.17, 1.32)	1.27 × 10^−11^

Model 1: Clinical risk factors only; model 2: GRS only; model 3: clinical risk factors and GRS.

**Figure 1:**
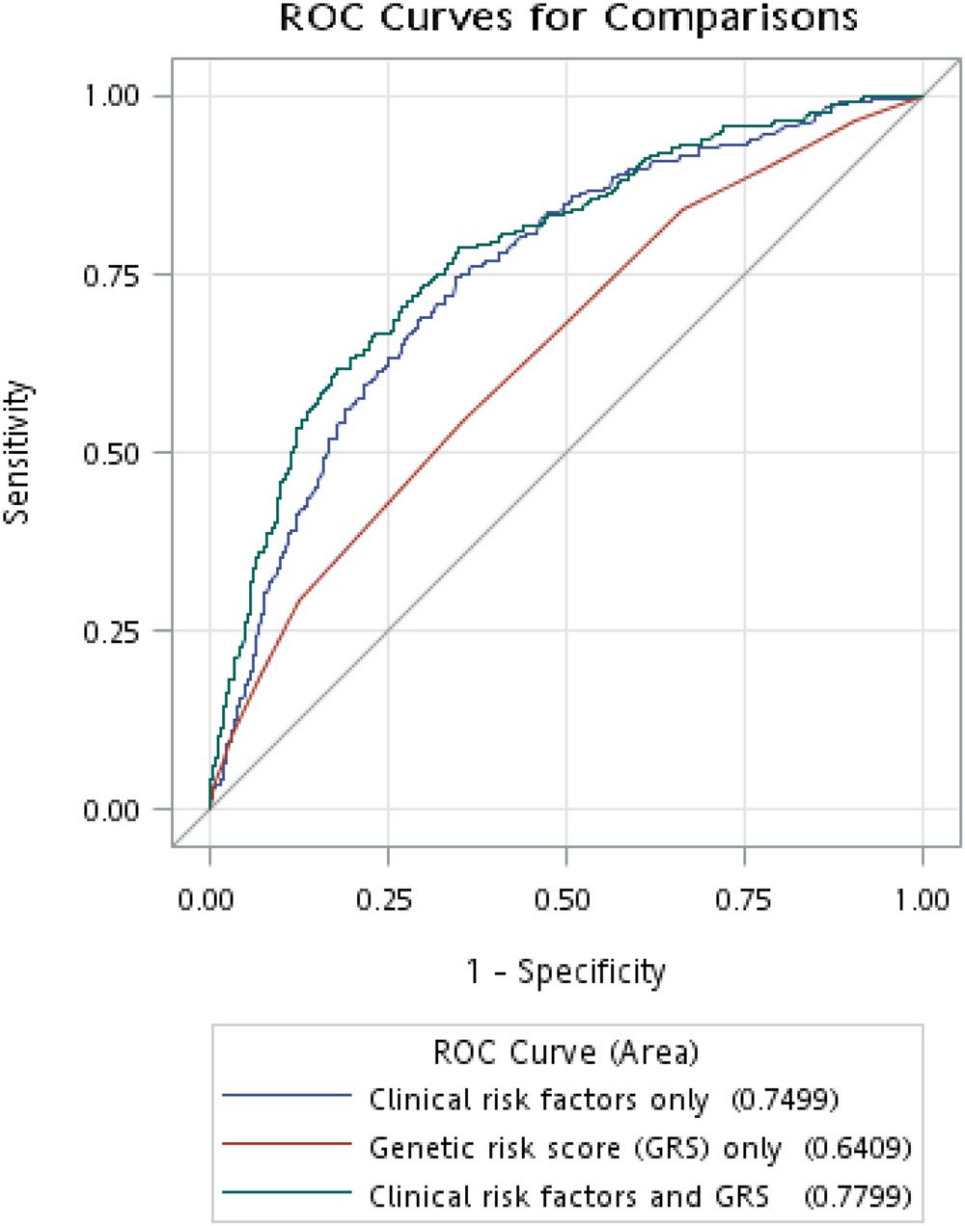
Areas under the receiver operating characteristics (AUROC) curve for DN status in derivation sample. The AUROC (95% confidence interval) for model 1 (clinical risk factors only), model 2 (GRS only), and model 3 (clinical risk factors and GRS) were 0.75 (0.72–0.78), 0.64 (0.60–0.68), and 0.78 (0.75–0.81), respectively. Model 1 did have better performance than model 2 (*P* = 5.12 × 10^−5^); and that were also found between models 1 and 3 (*P* = 0.002).

In the derivation set, the AUROC (95% CI) for model 1 (clinical risk factors only) was 0.75 (0.72–0.78), which was higher than that of model 2 (GRS only, 0.64 [0.60–0.68]) (Fig. 1). The addition of genetic information into the clinical risk factor model (model 3) increased the AUROC to 0.78 (0.75–0.81), *P* = 0.002, indicating that model 3 had better discrimination ability. Regarding results of using the wGRS, the prediction model had the same discrimination ability as that using GRS and the AUROC was 0.78 (0.75–0.81) (Supplement Fig. 1). Moreover, when BMI and triglycerides were treated as quantitative variables, the AUROC of model additionally adding GRS was 0.78 (0.75–0.81) (Supplement Fig. 2). In consideration of LD of two SNPs both in the same *RAE1* gene, we also constructed the new 6-SNP GRS by deleting one SNP at a time. Model 3 of using these two 6-SNP GRSs had the same discrimination ability (both AUROCs: 0.78, 95% CIs: 0.75–0.81) as the 7-SNP GRS, indicating that considering the other SNP cannot capture extra variation of the outcome and no problem arising from the collinearity on the study’s findings (Supplement Fig. 3A,B). In the external validation sample, the AUROCs for model 3 (additionally adding 7-SNP GRS or wGRS) were 0.70 (0.65–0.74) and 0.70 (0.66–0.75), respectively.

**Figure 2:**
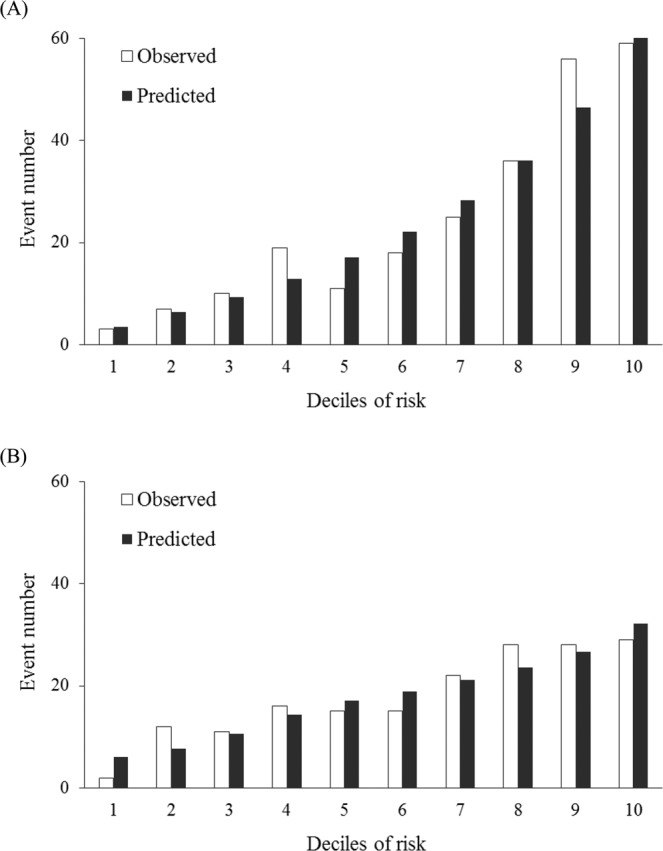
Predicted versus observed DN numbers according to the deciles of risk in (**A**) derivation (Hosmer–Lemeshow *χ*^2^ = 11.93, *P* = 0.155) and (**B**) validation samples (Hosmer–Lemeshow *χ*^2^ = 10.52, *P* = 0.230) by using the model with both clinical risk factors and GRS.

Calibration plots are presented in Fig. 2 for considering GRS and Supplement Fig. 4 for considering wGRS, showing the predicted versus observed DN numbers according to the deciles of risk in derivation and validation samples. The results of Hosmer–Lemeshow ${\chi }^{2}$ test revealed that the goodness of fit for our data was excellent (*P* = 0.155 and *P* = 0.230 in Fig. 2; and *P* = 0.394 and *P* = 0.299 in Supplement Fig. 4).

The calibration of the present model performance was assessed based on 1,000 samples from bootstrap resampling. The optimism corrected calibration intercept and corresponding slope were 0.01 (mean absolute error: 0.04) and 0.98 (mean absolute error: 0.12), respectively. The intercept was close to zero, indicating the absence of systematic deviation of the estimation of predicted probabilities. Moreover, the slope was close to one, indicating that the model was not overfitted. These statistics indicate a very good calibration for the present model. Furthermore, when the GRS was added to the risk prediction model of a set of clinical risk factors, the NRI was 9.98% (*P* = 0.001). The GRS did offer improvement in the performance of a DN model.

## Discussion

Our study established a DN risk prediction model including traditional clinical factors and genetic variants in a sample type 2 diabetes patients with and without DN. Moreover, this study validated the derived model in an external sample of the same characteristics. We derived a GRS by considering the risk allele for DN susceptibility SNPs based on our prior GWAS findings^31^. We identified significant demographic and clinical factors, including age, obesity, abnormal triglycerides, hypertension, and heart disease. The dominance of hypertension, obesity, and abnormal triglycerides in predicting risk is evident. This prediction model demonstrates that the highest predictive power for DN was observed when both clinical and genetic factors were considered with AUROCs of 0.78, which is higher than that when clinical risk factors were considered (0.75). Moreover, the addition of genetic factors to the clinical factors model resulted in a NRI of 9.98%. Based on our experience, we believe 9.98% for a NRI value is of clinical relevance. This prediction model enabled us to confirm the importance of GRS combined with clinical factors in predicting the risk of DN. Our validation results also showed good discrimination and calibration in the validation set. Thus, this model may be applied to identifying patients at a higher risk of DN to deliver interventions and appropriate DN prevention management.

Although many studies have established prediction models that combine clinical and genetic risk factors, few of them focus on kidney disease. Four published studies thus far have evaluated CKD prediction models, including one from Chinese type 2 diabetes patients^30^, two from a general population of European ancestry^27,28^, and one from the Japanese general population^29^. However, all authors did not report NRI values and they found that the creation of a GRS and its addition into the model with traditional risk factors did not substantially improve the discrimination of CKD risk. Due to the increases in C-statistic value were small in their studies, then, the values of NRI also were low. Moreover, they did not perform validation analysis.

Jiang *et al*. used the Hong Kong Diabetes Registry data from 2,755 type 2 diabetes patients and selected 36 SNPs (18 type 2 diabetes risk variants, 13 obesity risk variants, and 5 fasting plasma glucose risk variants; Supplement Table 3) to address the question for kidney disease^30^. These SNPs reached a genome-wide significance in European-origin populations with consistent replication in Chinese cohorts. To discover novel predictors of CKD, Jiang *et al*. repeatedly applied a stepwise selection based on the Akaike information criterion to subsamples of the cohort of 2,755 patients. As a result, they found that AUC was improved to 0.888 in the best clinical model, and the GRS score based on the top 3 SNPs improved the AUC to only 0.889 while adding GRS into the best clinical model. These selected clinical risk factors were age, ACR, eGFR, HbA1c, insulin, sensory neuropathy, ACEIs or ARBs, CHD, retinopathy, TG, and LDL. Moreover, the three selected genetic variants were rs478333 of *G6PC2* and rs7754840 and rs7756992 of *CDKAL1*.

O’Seaghdha *et al*. selected 16 SNPs (Supplement Table 3) that were associated with eGFR and stage 3 CKD from the CKDGen GWAS in European-origin population to construct a GRS^27^. Given the limited number of loci included in the GRS in their previous work, they selected 53 SNPs (Supplement Table 1) that are associated with lower eGFR from the recent CKDGen GWAS to construct a GRS^28^. Both O’Seaghdha *et al*. and Ma *et al*. used samples from the Framingham Heart Study and found that these GRS scores did not substantially improve discrimination of stage 3 CKD beyond the common clinical risk factors in general population (C statistics: 0.781 vs. 0.780 in O’Seaghdha’s study; 0.785 vs. 0.783 in Ma’s study). The clinical risk factors included age, sex, baseline eGFR, hypertension, diabetes, and proteinuria, which were identified from stepwise logistic regression^7^.

Fujii *et al*. combined 18 eGFR-associated SNPs (Supplement Table 3), which were identified from a GWAS into a GRS, and found that they were associated with CKD in a general Japanese population-based sample (n = 11,283) by using logistic regression analysis^29^. However, by adding the GRS into the clinical CKD risk factors (age, sex, hypertension, and type 2 diabetes) model, they found that the improvement of discriminatory ability of CKD prevalence was small. The C statistic was 0.720 in the model considering traditional covariates along with the GRS and 0.719 in the model with traditional covariates.

In this study, we used the GRS, which is comprised of genetic variants that were already there at birth, and a set of clinical risk factors, which were observed at the time of DN observation, to predict DN. Our results show the AUROC (95% CI) for model with clinical risk factors only, as well as model with GRS only were 0.75 (0.72–0.78) and 0.64 (0.60–0.68), respectively. Then, the prediction model with both genetic and environmental factors have an AUROC value of 0.78 (0.75–0.81). Furthermore, we calculated the NRI to carry out an evaluation of our models with and without genetic factors, and that was 9.98%. The GRS did offer improvement in the performance of our DN model, i.e., confirming the importance of GRS-based predictor integrated into prediction model of common clinical risk factors for personalized DN risk prediction. Our prediction model provides new insights for genetic screening test to identify patients at high risks for DN that disease prevention intervention could be targeted at. Moreover, through the prediction model of DN, patients and clinical staffs can easily understand the individuals’ risk factors and levels of DN.

There may be some possible limitations in this study. First, our DN genetic risk model was performed in a Han Chinese population, and the model was not probably applicable to all populations. The second limitation concerns our study samples from only one site. Further studies would be necessary to validate our results in Han Chinese population with type 2 diabetes. Finally, due to use of different genotyping platforms in our validation sample, the GRS was built using both observed and imputed data. Although that may have misclassification of genotypes by introducing information error, resulting in diluting the strength of the relationship between GRS and DN status, which is a lesser threat to validity.

In summary, we have constructed a GRS based on SNPs from our prior GWAS findings and demonstrated that the addition of genetic information into the conventional risk factor model could offer improvement on the DN risk prediction in Han Chinese type 2 diabetes patients. Moreover, our validation results show good discrimination and calibration. This prediction model enabled us to confirm the importance of GRS combined with clinical factors in predicting the risk of DN and may be applied to identifying high-risk patients of DN in order to provide interventions and appropriate DN prevention management.

## Materials and Methods

### Study individuals

In the current study, a case–control study design was used for both derivation and validation samples. Individuals diagnosed with type 2 diabetes were included based on the American Diabetes Association (ICD-9-CM code: 250) criteria for diagnosis of type 2 diabetes. We excluded individuals with type 1 diabetes (ICD-9-CM codes: 250.x1/x3), gestational diabetes (ICD-9-CM codes: 648.83), and maturity-onset diabetes of the young. Diabetic patients with eGFR <60 mL/min/1.73 m^2^ or proteinuria as determined through a spot urine dipstick of >1+ were defined as DN cases^31^, and patients without nephropathy were defined as diabetic controls. In the derivation stage, in order to maximize our sample size, we used all 995 type 2 diabetes patients (246 DN cases and 749 diabetic controls) in our previous GWAS study^31^, and they were recruited from China Medical University Hospital (CMUH). In the validation stage, an additional independent sample consisting of 179 DN cases from clinical setting and 340 diabetic controls from the community setting was used and genotyped during the period 2014 to 2015. These DN cases from the nephrology clinic in CMUH and diabetic controls who attended a 1-day health check in CMUH were recruited. All participants were of Han Chinese origin, including Minnan, Hakka, and Mainland Chinese. All patients signed informed consent forms. This study was approved by the Human Research Committee of China Medical University Hospital and all methods were performed in accordance with the relevant guidelines and regulations.

### Measurements

Self-administered questionnaires were utilized for each subject to collect data, including sociodemographic and lifestyle characteristics (including current smoking status [self-reported yes/no] and alcohol drinking [self-reported yes/no]), as well as self-reported health status. Hypertension was defined as undergoing treatment for elevated blood pressure or self-reported. Both heart disease and cerebral vascular accident (CVA) were defined as the use of medications or self-reported. Duration of diabetes (years) was defined as the time from diagnosis to enrollment in the study. The body mass index (BMI) was calculated as weight divided by height squared (kg/m^2^); moreover, obesity was defined as BMI ≥27 kg/m^2^. After a 12 h overnight fasting, blood samples were taken in the morning. We also collected spot morning urine samples. Total cholesterol, triglycerides, low-density lipoprotein cholesterol (LDL-C), high-density lipoprotein cholesterol (HDL-C), creatinine, uric acid, and blood urea nitrogen (BUN) were analyzed by the Synchron LX20 system (Beckman Coulter, Synchron LX20, Fullerton, CA, USA). Hemoglobin A1c (HbA1c) testing was also performed. To assess the renal function of a patient, we used the Modification of Diet in Renal Disease study equation for Taiwanese: eGFR (ml/min/1.73 m^2^) = 175 × [serum creatinine (mg/dL)^−1.154^ × (age)^−0.203^ × (0.742 if female) × 0.945]^31,32^. In addition, we used a spot urine dipstick test to detect proteinuria, which was defined as a positive dipstick test (>1+)^33^. Based on the report of American Diabetes Association^34^, the following variables were considered as clinical risk factors of DN: age, gender, smoking status, alcohol drinking, duration of diabetes, obesity, HbA1c, total cholesterol, triglycerides, LDL-C, HDL-C, hypertension, heart disease, and CVA.

### SNPs selection and genotyping

From our previous GWAS findings^31^, we selected the seven SNPs that were identified and associated with DN in a Han Chinese population with type 2 diabetes. These DN susceptibility SNPs include rs10963767 (*ADAMTSL1*), rs11647932 (*ST3GAL2*), rs11645214 (*SF3B3*), rs6499323 (*IL34*), rs182784 (*BMP7*), rs4811839 (*RAE1*), and rs6025517 (*RAE1*). For genotyping analysis, genomic DNA was isolated from the blood samples. In the derivation set, 995 type 2 diabetes patients were genotyped using Illumina HumanHap550-Duo BeadChip, which was performed by deCODE Genetics (Reykjavík, Iceland). In the validation set, DNA samples from 340 type 2 diabetes patients were genotyped using an Illumina VeraCode GoldenGate genotyping assay (Illumina, San Diego, CA, USA), including the 7 considered SNPs. DNA samples from 179 DN cases were genotyped using custom Taiwan Biobank chips (TWB chip) and run on the Axiom genome-wide array plate system (Affymetrix, Santa Clara, CA, USA). Due to use of different genotyping platforms, genotype imputation was performed using the IMPUTE2 software^35^ in DN cases from our validation sample. Genotype imputation refers to the statistical inference of unobserved genotypes. It includes two steps: first, inferring the haplotypes in a study dataset; second, combining the inferred haplotypes with the haplotypes of a genotyped reference panel to fill in unobserved genotypes in a study dataset. The reference panel from 1,000 Genomes Project was used. The GRS in DN cases from our validation sample was built using both observed and imputed data. Each SNP was tested for deviation from the Hardy–Weinberg equilibrium (HWE) using exact tests of HWE in PLINK (v1.07).

### Statistical analysis

The demographic and clinical characteristics of study subjects were examined. For continuous variables, the mean ± standard deviation were reported. For categorical variables, the number and percentage of observations were reported. In the bivariate analyses, we performed two-sample t-test and Chi-square test. Seven SNPs including rs10963767 (minor allele C), rs11647932 (T), rs11645214 (G), rs6499323 (G), rs182784 (G), rs4811839 (G), and rs6025517 (C) were selected to define a person’s individual genetic risk for DN based on our prior GWAS findings (the discovery GWAS)^31^. The unweighted GRS was constructed for each individual by summing the number of risk alleles (coded as 0, 1, and 2) carried. The effects of these risk alleles from the derivation set were consistent with those identified in the discovery GWAS^31^. For the weighted GRS (wGRS), we used summary statistics from the discovery GWAS^31^, and it was defined as a weighted sum of the number of risk alleles of these seven considered SNPs.

In the derivation set, three predictive models were fitted to the data, in which the DN status was a function of (1) clinical risk factors only, (2) GRS (or wGRS) only, and (3) clinical risk factors and GRS (or wGRS) by using logistic regression models. To develop the best prediction model of DN^36^, we performed the following: (1) univariable analysis for each independent variable; (2) selection of independent variable with univariable test of a *P*-value < 0.25^37,38^ as a candidate predictor for our multivariable model; (3) construction of a multivariable model with these candidate predictors without collinearity and backward elimination procedure of selected predictors reaching significance of 0.05. Moreover, when age and gender were not statistically significant to be candidates for the multivariable model, we forced them into the final model. The strength of association between risk factors and DN was measured by odds ratios (ORs) and their 95% confidence intervals (CIs). In the validation set, we included the same parameters in the validation model that estimated their values (i.e. weights) within the model itself.

The predictive performance of the DN risk prediction model (both discrimination and calibration) was evaluated. The predictive models’ ability to discriminate DN status was evaluated by the areas under the receiver operating characteristics (AUROC) curve. We performed the Hosmer–Lemeshow goodness-of-fit test to compare the observed and predicted events of DN, and patients were grouped by decile of predicted probability. Furthermore, calibration was carried out to correct the potential for overfitting by using 1,000 times bootstrap resampling^39^, and model calibration was conducted by using calibration-in-the-large and calibration slope approaches. To assess how well a new model correctly reclassifies subjects as compared to an old model, the net reclassification improvement (NRI) was introduced^40^. In our study, we calculated the NRI to quantify improvement in prediction performance gained by adding the GRS to a set of clinical risk factors for predicting DN. When we calculated the NRI, we combined the two samples and adopted the parameter values estimated from the derivation sample. Tertile cut-off points were used to categorize DN risk into low-, medium-, and high-risk groups. We performed statistical analysis using SAS 9.4 (SAS Institute Inc, Cary, NC, USA) and PLINK (v1.07). Statistical significance was considered at a two-sided *P*-value < 0.05.

## Supplementary information

Supplement Tables and Figures

## Data Availability

All data generated or analysed during this study are included in this published article.
