# Supplementary material for: Genetic risk score for risk prediction of diabetic nephropathy in Han Chinese type 2 diabetes patients
Source: Sci Rep. 2019 Dec 27;9:19897. doi: 10.1038/s41598-019-56400-3 (PMC6934611; doi:10.1038/s41598-019-56400-3)
Supplement: Supplementary file 1 — Supplement Tables and Figures [file 41598_2019_56400_MOESM1_ESM.docx]

**Genetic risk score for risk prediction of diabetic nephropathy in Han Chinese type 2 diabetes patients**

Li-Na Liao^1^, Tsai-Chung Li^1,2^, Chia-Ing Li^3,4^, Chiu-Shong Liu^3,4,5^, Wen-Yuan Lin^3,5^, Chih-Hsueh Lin^3,5^, Chuan-Wei Yang^4^, Ching-Chu Chen^6,7^, Chiz-Tzung Chang^3,8^, Ya-Fei Yang^3,8^, Yao-Lung Liu^3,8^, Huey-Liang Kuo^3,8,9^, Fuu-Jen Tsai^7,10,*^, and Cheng-Chieh Lin^3,4,5,*^

1. Department of Public Health, College of Public Health, China Medical University, Taichung, Taiwan
2. Department of Healthcare Administration, College of Medical and Health Sciences, Asia University, Taichung, Taiwan
3. School of Medicine, College of Medicine, China Medical University, Taichung, Taiwan
4. Department of Medical Research, China Medical University Hospital, Taichung, Taiwan
5. Department of Family Medicine, China Medical University Hospital, Taichung, Taiwan
6. Division of Endocrinology and Metabolism, Department of Medicine, China Medical University Hospital, Taichung, Taiwan
7. School of Chinese Medicine, College of Chinese Medicine, China Medical University, Taichung, Taiwan
8. Kidney Institute and Division of Nephrology, Department of Internal Medicine, China Medical University Hospital, Taichung, Taiwan.
9. Graduate Institute of Clinical Medical Science, College of Medicine, China Medical University, Taichung, Taiwan.
10. Human Genetic Laboratory, Department of Medical Research, China Medical University Hospital, Taichung, Taiwan.

***Corresponding author:** Cheng-Chieh Lin

China Medical University, No. 91, Hsueh-Shih Road, Taichung, 40421, Taiwan

Tel: 886-4-2205-3366 ext. 1013, Fax: 886-4-2207-8539

E-mail: [cclin@mail.cmuh.org.tw](mailto:cclin@mail.cmuh.org.tw)

***Corresponding author:** Fuu-Jen Tsai

E-mail: [d0704@mail.cmuh.org.tw](mailto:d0704@mail.cmuh.org.tw)

Supplement Table 1. ORs and their 95% CIs for diabetic nephropathy in derivation sample by using the wGSR as predictor

| Variable | Model 1 | |  | Model 2 | |  | Model 3 | |
| --- | --- | --- | --- | --- | --- | --- | --- | --- |
|  | OR (95% CI) | P-value |  | OR (95% CI) | P-value |  | OR (95% CI) | P-value |
| Gender (ref. women) | 1.11 (0.81, 1.51) | 0.529 |  | － | － |  | 1.14 (0.82, 1.57) | 0.434 |
| Age (years) | 1.07 (1.05, 1.09) | <0.01×10^-12^ |  | － | － |  | 1.08 (1.06, 1.10) | <0.01×10^-12^ |
| Obesity (ref. BMI<27 kg/m^2^) | 1.59 (1.14, 2.22) | 0.007 |  | － | － |  | 1.62 (1.14, 2.29) | 0.007 |
| Abnormal triglycerides  (ref. <150 mg/dL) | 1.63 (1.19, 2.24) | 0.002 |  | － | － |  | 1.56 (1.13, 2.17) | 0.008 |
| Hypertension (ref. No) | 2.03 (1.46, 2.81) | 2.26×10^-5^ |  | － | － |  | 2.12 (1.51, 2.98) | 1.32×10^-5^ |
| Heart disease (ref. No) | 1.56 (1.08, 2.26) | 0.018 |  | － | － |  | 1.47 (1.00, 2.17) | 0.048 |
| wGRS | － | － |  | 1.42 (1.28, 1.56) | 6.20×10^-12^ |  | 1.46 (1.31, 1.63) | 8.89×10^-12^ |

Model 1: Clinical risk factors only; model 2: wGRS only; model 3: clinical risk factors and wGRS.

Supplement Table 2. ORs and their 95% CIs for diabetic nephropathy in derivation sample by using BMI and triglycerides as quantitative variables

| Variable | Model 1 | |  | Model 2 | |  | Model 3 | |
| --- | --- | --- | --- | --- | --- | --- | --- | --- |
|  | OR (95% CI) | P-value |  | OR (95% CI) | P-value |  | OR (95% CI) | P-value |
| Gender (ref. women) | 1.07 (0.78, 1.46) | 0.683 |  | － | － |  | 1.10 (0.79, 1.52) | 0.574 |
| Age (years) | 1.07 (1.05, 1.09) | <0.01×10^-12^ |  | － | － |  | 1.08 (1.06, 1.10) | <0.01×10^-12^ |
| BMI (kg/m^2^) | 1.05 (1.01, 1.10) | 0.027 |  | － | － |  | 1.04 (1.00, 1.09) | 0.058 |
| Triglycerides (mg/dL) | 1.00 (1.00, 1.00) | 0.001 |  | － | － |  | 1.00 (1.00, 1.00) | 0.001 |
| Hypertension (ref. No) | 1.94 (1.40, 2.70) | 8.10×10^-5^ |  | － | － |  | 2.04 (1.45, 2.87) | 4.14×10^-5^ |
| Heart disease (ref. No) | 1.57 (1.09, 2.28) | 0.017 |  | － | － |  | 1.51 (1.02, 2.22) | 0.037 |
| GSR | － | － |  | 1.22 (1.15, 1.29) | 9.24×10^-12^ |  | 1.24 (1.17, 1.32) | 1.29×10^-11^ |

Model 1: Clinical risk factors only; model 2: GRS only; model 3: clinical risk factors and GRS.

Supplement Table 3. Selected SNPs list and C-statistics

| Author | Country  (Ethnicity) | Outcome | Study design | Study subjects | Clinical risk factors model | | Genetic risk factors model | | | Combined model | | Selected SNPs |
| --- | --- | --- | --- | --- | --- | --- | --- | --- | --- | --- | --- | --- |
|  |  |  |  |  | Variables | C-statistic | No. SNPs | C-statistic | OR (95% CI) per allele of GRS | C-statistic | OR (95% CI) per allele of GRS |  |
| Jiang et al., 2016 | China (Chinese) | CKD (ICD 9 codes) | Cohort study | Type 2 diabetes patients (Hong Kong diabetes registry; n=2,755) | Age, ACR, eGFR, HbA1c, insulin, sensory neuropathy, ACEIs or ARBs, CHD, retinopathy, TG, and LDL. | AUC 0.888 | 18 type 2 diabetes, 13 obesity, and 5 glucose SNPs | NR | NR | AUC 0.889 | HR 1.17 (1.10-1.26) | 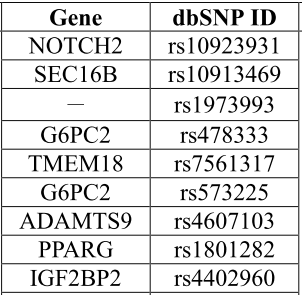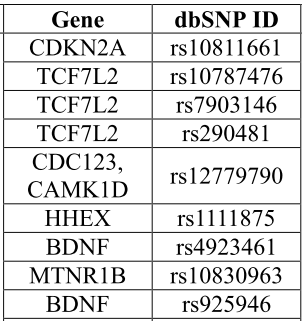  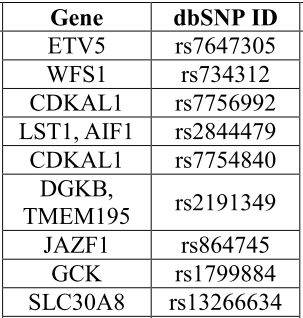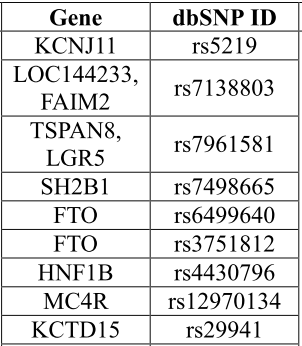 |
|  |  |  |  |  |  |  |  |  |  | Finally, calculated a GRS based on the top 3 SNPs. | |  |
| O’Seaghdha et al., 2012 | US (European ancestry) | CKD (eGFR<60) | Cohort study | General population (Framingham heart study; n=2,489) | Age, sex, cohort status, baseline eGFR, hypertension, diabetes, and proteinuria. | 0.780 | 16 eGFR SNPs | NR | 1.06 (1.01-1.11)^a^ | 0.781 | 1.05 (1.00-1.11) | 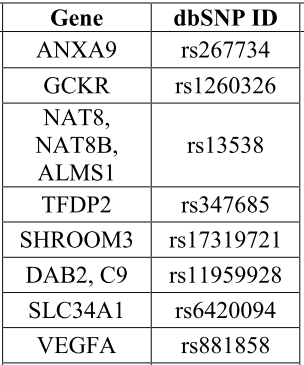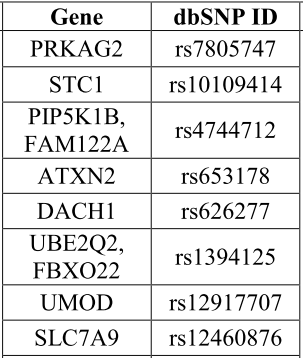 |
| Ma et al., 2017 | US (European ancestry) | CKD (eGFR<60) | Cohort study | General population  (Framingham heart study; n=2,698) | Age, sex, cohort status, baseline eGFR, hypertension, diabetes, proteinuria. | 0.783 | 53 eGFR SNPs | NR | 1.44 (1.08-1.93)^b^ per 10 alleles of GRS | 0.785 | 1.37 (1.02-1.83) per 10 alleles of GRS | 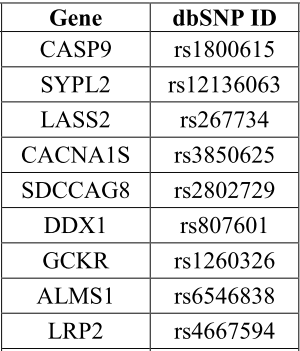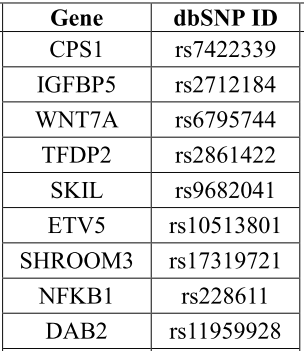  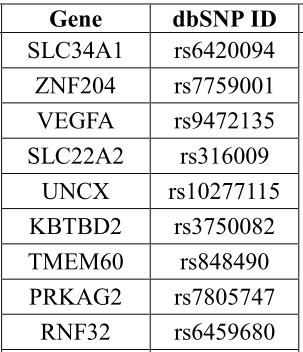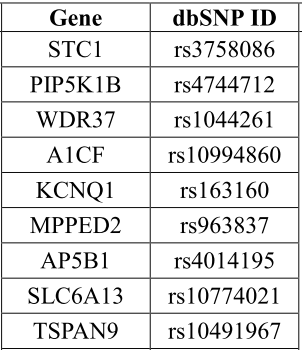  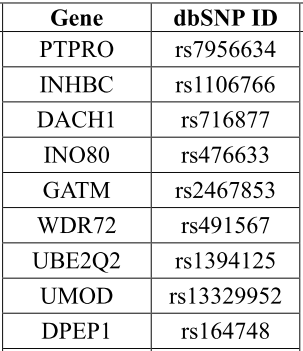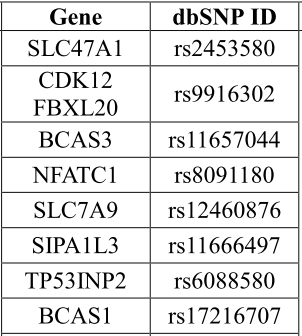 |
| Fujii et al., 2018 | Japan (Japanese) | CKD (eGFR<60) | Cohort study | General population  (n=11,283) | Age, sex, type 2 diabetes, and hypertension. | 0.719 | 18 eGFR SNPs | NR | 1.125 (1.049-1.206) per 10 GRS increment | 0.720 | 1.120 (1.042-1.203) per 10 GRS increment | 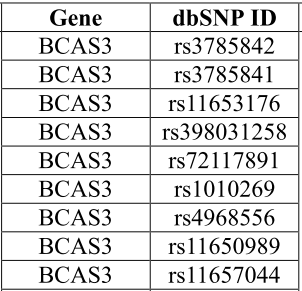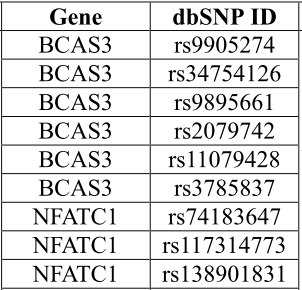 |

a: Age and sex adjusted. b: Age, sex, cohort status. NR: not reported.


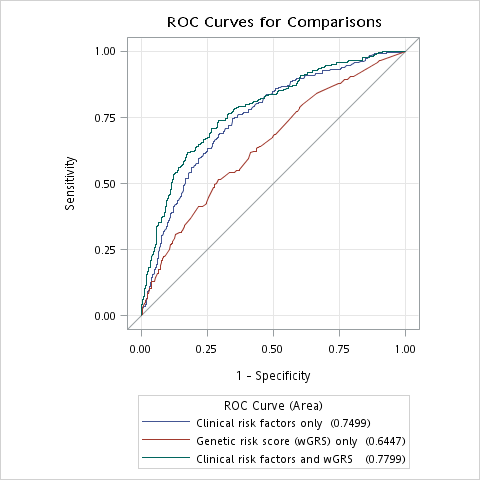


Supplement Figure 1. Areas under the receiver operating characteristics (AUROC) curve for DN status in derivation sample. The AUROC (95% confidence interval) for model 1 (clinical risk factors only), model 2 (wGRS only), and model 3 (clinical risk factors and wGRS) were 0.75 (0.72–0.78), 0.64 (0.60–0.68), and 0.78 (0.75–0.81), respectively. Model 1 did have better performance than model 2 (*P*=9.97×10^-5^); and that were also found between models 1 and 3 (*P*=0.002).


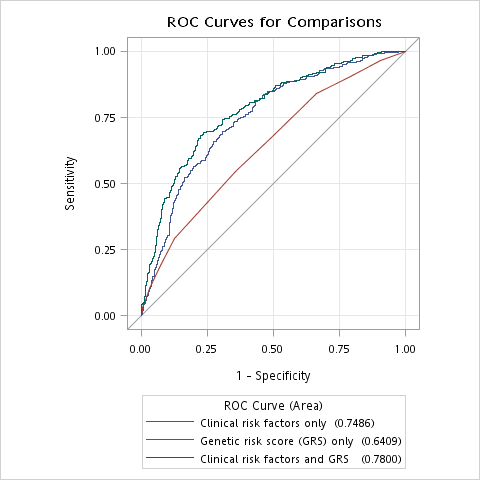


Supplement Figure 2. AUROC curve for DN status in derivation sample when BMI and triglycerides were treated as quantitative variables. The AUROC (95% CI) for model 1 (clinical risk factors only), model 2 (GRS only), and model 3 (clinical risk factors and GRS) were 0.75 (0.71–0.78), 0.64 (0.60–0.68), and 0.78 (0.75–0.81), respectively. Model 1 did have better performance than model 2 (*P*=6.35×10^-5^); and that were also found between models 1 and 3 (*P*=0.002).


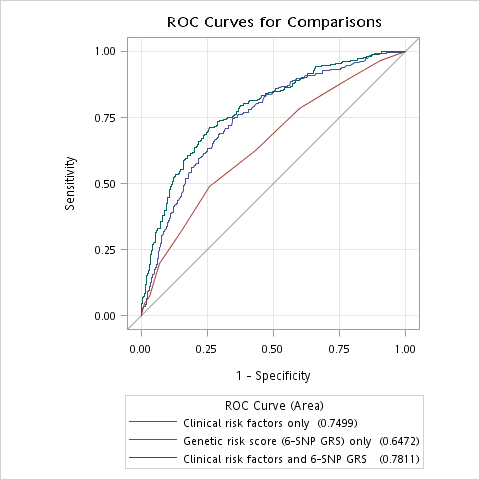


(A) 6-SNP GSR, no including rs6025517


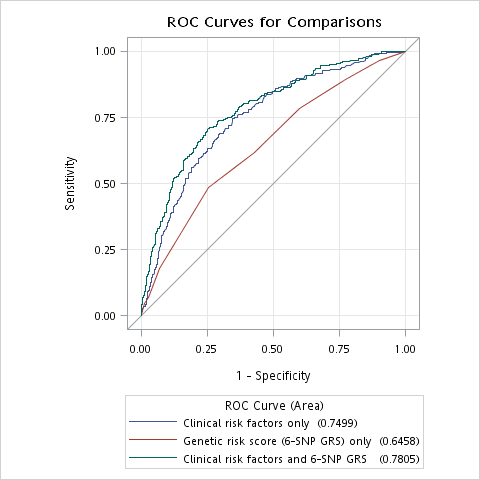


(B) 6-SNP GSR, no including rs4811839

Supplement Figure 3. AUROC curve for DN status in derivation sample when using 6-SNP GRS.
(A) When rs6025517 was not included, the AUROC (95% CI) for model 1 (clinical risk factors only), model 2 (6-SNP GRS only), and model 3 (clinical risk factors and 6-SNP GRS) were 0.75 (0.72–0.78), 0.65 (0.61–0.69), and 0.78 (0.75–0.81), respectively. Model 1 did have better performance than model 2 (*P*=1.42×10^-4^); and that were also found between models 1 and 3 (*P*=0.002).
(B) When rs4811839 was not included, the AUROC (95% CI) for model 1 (clinical risk factors only), model 2 (6-SNP GRS only), and model 3 (clinical risk factors and 6-SNP GRS) were 0.75 (0.72–0.78), 0.66 (0.61–0.69), and 0.78 (0.75–0.81), respectively. Model 1 did have better performance than model 2 (*P*=1.08×10^-4^); and that were also found between models 1 and 3 (*P*=0.002).


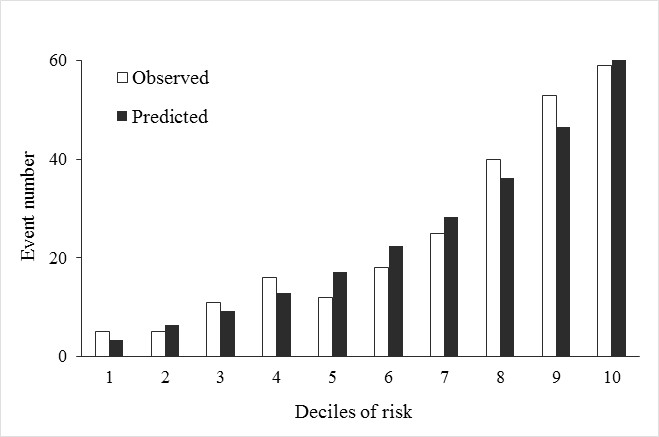


(A)


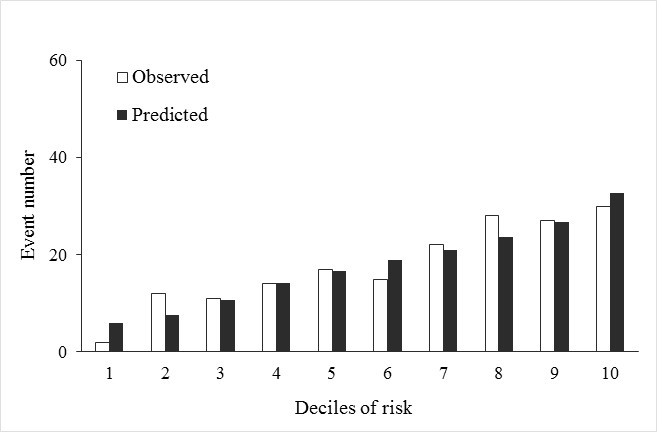


(B)

Supplement Figure 4. Predicted versus observed DN numbers according to the deciles of risk in (A) derivation (Hosmer–Lemeshow $\chi^{2}$=8.41, *P*=0.394) and (B) validation samples (Hosmer–Lemeshow $\chi^{2}$=9.54, *P*=0.299) by using the model with both clinical risk factors and wGRS.
